# Alternative Wood Raw Material Sources in Particleboard and OSB Production—Challenges and Perspectives

**DOI:** 10.3390/polym17131760

**Published:** 2025-06-26

**Authors:** Dorota Dukarska, Jakub Kawalerczyk, Ján Sedliačik, Petar Antov, Mehr Unisa

**Affiliations:** 1 Department of Mechanical Wood Technology, Faculty of Forestry and Wood Technology, Poznań University of Life Sciences, Wojska Polskiego 38/42, 60-627 Poznań, Poland; 2 Department of Furniture and Wood Products, Technical University in Zvolen, T.G. Masaryka 24, 960 01 Zvolen, Slovakia; jan.sedliacik@tuzvo.sk; 3 Department of Mechanical Wood Technology, Faculty of Forest Industry, University of Forestry, 1797 Sofia, Bulgaria; p.antov@ltu.bg; 4 Independent Researcher, 80230 Joensuu, Finland; mehernisa000@gmail.com

**Keywords:** particleboard, oriented strand board, raw material, wood composites, juvenile wood, fast-growing wood, wood waste, recycling, sustainable production

## Abstract

This review examines the potential use of alternative wood raw materials, including fast-growing plantation species, juvenile wood, non-plantation species, and recycled wood, in the production of particleboard (PB) and oriented strand board (OSB). In light of the ongoing challenges faced by the wood-based industry in securing a stable and sustainable supply of raw materials, these alternatives present several advantages, such as cost-effectiveness, greater availability, and reduced reliance on natural forest resources. Fast-growing plantation species and juvenile wood are particularly suited for lightweight applications, while non-plantation species and recycled wood contribute to sustainability goals by lowering environmental impact and promoting resource efficiency. Nonetheless, the successful integration of these materials requires overcoming certain challenges, including variability in their physical and mechanical properties, as well as the need for tailored adhesive systems and processing parameters. This review examines strategies to optimize production processes and enhance the utilization of waste materials while emphasizing the role of alternative raw materials in advancing circular economy principles. The findings highlight the importance of future research to improve material knowledge, technological solutions, and industry practices, thereby supporting the sustainable development of the wood-based materials sector.

## 1. Introduction

**Figure 1:**
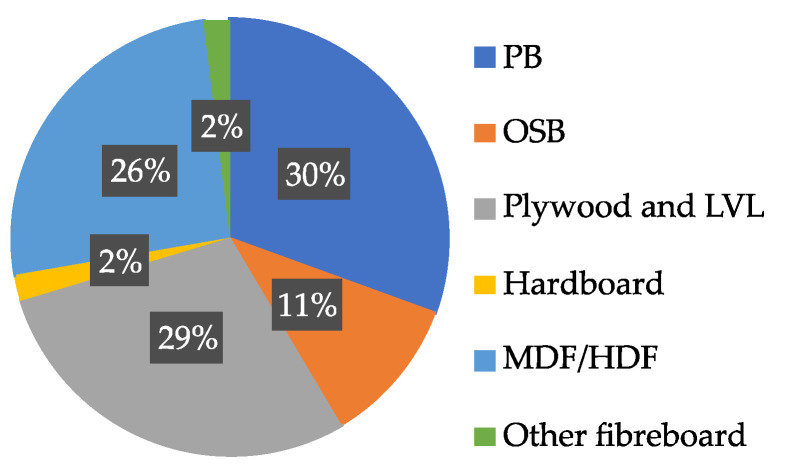
Production structure of selected wood-based boards worldwide in 2023 according to FAOSTAT [1].

Wood-based materials play a crucial role in the furniture, construction, and interior finishing industries, offering a versatile and sustainable alternative to solid wood. Among these materials, several major groups can be distinguished, including particleboards (PBs), oriented strand boards (OSBs), medium-density and high-density fibreboards (MDF, HDF), as well as plywood. These wood-based boards differ significantly in terms of raw material composition and mechanical properties. As shown in Figure 1 prepared based on data from the Statistical Database of the Food and Agriculture Organization of the United Nations (FAOSTAT), particleboards are among the most widely used [1] primarily due to their versatility, favorable physical and mechanical characteristics, cost-effectiveness, and the possibility of surface enhancement using various techniques. Moreover, the wide range of raw materials suitable for their production makes them one of the most commonly used wood-based materials. OSBs are also of particular importance due to their high strength, resistance to environmental conditions, and growing use in the construction sector, where they are widely applied in both structural and finishing elements of various building projects. According to the European Panel Federation, in 2023, approximately 66% of all particleboards produced were used in furniture manufacturing, 27% in construction, and 1% in packaging production, with the remainder utilized across various other industrial sectors [2].

**Figure 2:**
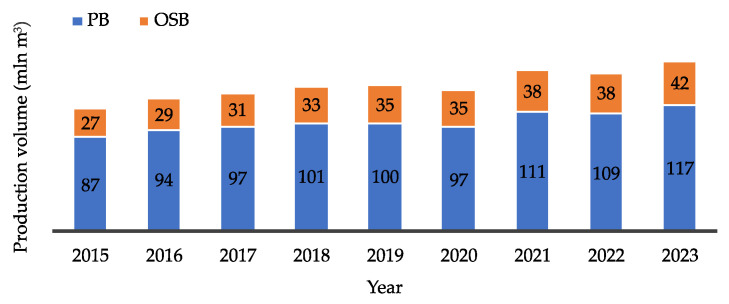
Volume of PB production from 2015 to 2023 as reported by FAOSTAT [2].

**Figure 3:**
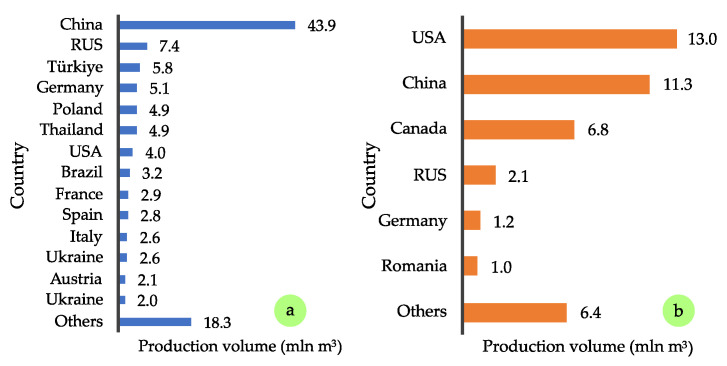
The world’s largest manufacturers of (**a**)—PBs, (**b**)—OSBs [2].

**Figure 4:**
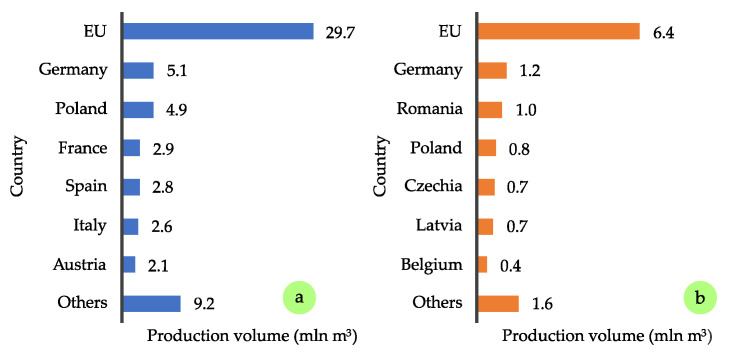
Production volumes of (**a**) PBs, (**b**) OSBs in the European Union (EU) and in the member countries with the highest production levels [2].

For these reasons, production volumes have been constantly increasing, as illustrated in Figure 2. According to FAOSTAT reports [1], global PBs production rose from 87.3 million m^3^ in 2015 to 116.6 million m^3^ in 2023, an increase of approximately 34%, which highlights the dynamic growth of this sector in recent years. An even more substantial rise was recorded for OSB production during the same period, increasing from 27 million m^3^ to 42 million m^3^, representing a 56% growth. The leading producers of PB include China, with an annual production of approximately 44 million m^3^, followed by Russia (7.4 million m^3^), and countries such as Turkey, Germany, Poland, and Thailand, each producing around 5 million m^3^ (Figure 3a). In terms of OSB production, the top producers are the United States (13.0 million m^3^), China (11.3 million m^3^), and Canada (6.8 million m^3^) (Figure 3b). It is also valuable to assess the situation in the European market, particularly within the European Union (EU). Across the EU, total PB production is estimated at 29.7 million m^3^ (Figure 4a). Germany and Poland are the leading producers within the EU, each reaching approximately 5 million m^3^ annually, followed by France with 2.9 million m^3^ [2]. As for OSB (Figure 4b), the highest outputs are recorded in Germany (1.2 million m^3^), Romania (1.0 million m^3^), and Poland (0.8 million m^3^).

Wood raw material remains the fundamental component in PB production, accounting for approximately 84.5% of total input in 2023 [3]. Traditionally, the primary raw material used in the production of PB and OSB boards has been softwood sourced directly from forests. In the case of PBs, lower-grade wood obtained from primary wood processing, such as chips, shavings, and sawdust, is also widely used. In contrast, the use of hardwood in the manufacture of these boards has been less common, with relatively limited research and industrial application reported in the literature [4].

**Figure 5:**
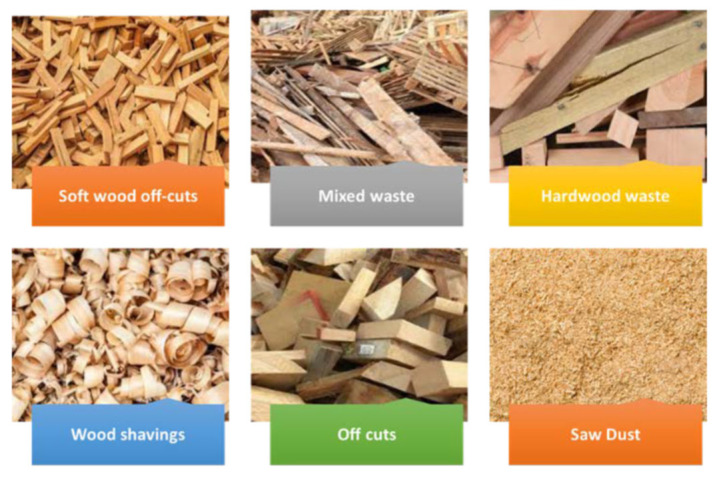
Various forms of wood waste. Adapted from Jahan et al. [5] (open access).

**Figure 6:**
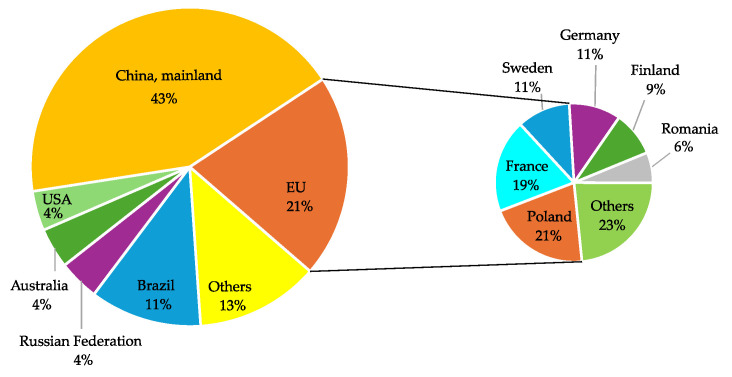
Global and EU wood waste production by FAOSTAT w 2023 r. [2].

In recent years, however, recycled wood has gained increasing importance as a supplementary raw material. Leading PB manufacturers have long incorporated recycled wood sources, including construction and demolition waste, discarded furniture, transport pallets, and other wood-based elements (Figure 5). It is estimated that approximately 250 million m^3^ of wood waste is generated globally each year, with around 50 million m^3^ originating from EU countries, representing 21% of the total (Figure 6) [2].

**Figure 7:**
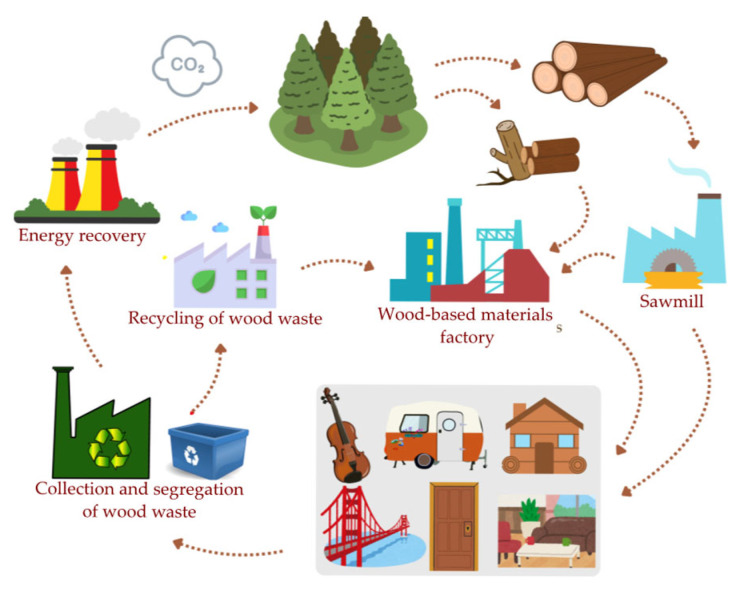
Cascade use of wood.

This trend aligns with the principles of sustainable development and the circular economy, helping to reduce the demand for primary raw materials and divert wood waste from landfills. Recently, the European Parliament approved the intensification of cascaded wood use as a strategy to promote circularity and mitigate climate change (Figure 7) [6]. Cascaded use refers to the efficient management of resources through the repeated utilization of residues and recycled materials, thereby increasing the overall availability of biomass within a given system [7]. However, effective recycling in this sector requires advanced technologies for sorting and processing secondary raw materials to ensure the quality and purity necessary for further manufacturing. Despite these challenges, PB production is considered highly resource-efficient, as wood waste can be transformed into value-added products. This is particularly important given the growing demand for wood raw materials across the wood-based material, paper, and energy industries, alongside the increasing scarcity and rising costs of harvested wood. Unfortunately, due to the specific production requirements and strand geometry of OSBs, they are currently manufactured exclusively from harvested wood rather than recycled sources [8].

For many years, extensive research has been conducted on the potential use of alternative lignocellulosic raw materials, particularly agricultural biomass. Numerous studies have demonstrated the feasibility of substituting wood particles, either partially or entirely, in PB production with materials such as rapeseed, wheat, mustard, and rice straw [9,10,11,12,13,14,15], stalks of sunflower, Jerusalem artichoke, cup-plant, cotton, corn, tobacco, chili peppers, and eggplant [16,17,18,19,20,21,22], husks of oat, barley, corn, rice, coffee, and castor [23,24,25,26,27], sugarcane bagasse [27]; residues from hemp and horse chestnut seeds [28,29,30]; leaves of sugarcane, tea, and sycamore [31,32,33], elephant grass, Miscanthus, reed canary grass, and bamboo [34,35,36,37]; and even shells of almonds, walnuts, hazelnuts, and peanuts [38,39,40,41]. Investigations have also explored the application of non-wood lignocellulosic materials in OSB manufacturing, such as wheat straw [42,43] and bamboo [44]. Despite the promising potential, industrial implementation of such raw materials presents several challenges. Most notably, these alternative sources exhibit significant variability in their chemical composition and physical properties, which complicates the production of boards with uniform performance. They also tend to contain higher levels of extractives, lignin, and phenolic compounds, which can negatively affect adhesive interactions and weaken internal bonding. Moreover, these materials are generally more susceptible to biodegradation and microbial activity. Their seasonal availability and dependence on harvest cycles pose further constraints, making continuous production difficult and necessitating large-scale storage, which in turn introduces additional costs and risks of degradation. Processing these non-wood materials through size reduction, drying, and in some cases chemical modification may also increase production costs and require adjustments to standard board manufacturing technologies. For these reasons, the search continues for alternative wood-based raw materials that offer better compatibility with existing industrial processes, enhanced product performance, and greater production efficiency. In addition, wood as a renewable and recyclable resource supports the principles of circular economy by facilitating resource efficiency, enabling material recovery, and minimizing waste throughout the production cycle.

This article provides a comprehensive review of the potential use of various alternative wood raw materials in the production of PB and OSB boards, with a particular focus of their species and origin on final product quality and the sustainable development of the wood-based materials industry.

## 2. Innovative Approaches to Raw Material Selection in Wood-Based Material Manufacturing

In response to the growing demand for particleboards and the increasing need to optimize raw material management, considerable attention is now being directed toward alternative sources of wood material. Numerous studies have shown that, in addition to conventional sources, juvenile wood, fast-growing species, recycled wood waste, and forestry by-products can also be utilized in the production of PB and OSB. The incorporation of these raw materials into industrial practice requires the adjustment of processing technologies, optimization of gluing and pressing parameters, and the development of methods aimed at improving the uniformity and stability of the final product properties. The following sections of this paper provide an overview of the most relevant groups of alternative raw materials, their role in board production, and the necessary adaptations in manufacturing conditions to ensure their effective and sustainable use.

### 2.1. Juvenile Wood

**Figure 8:**
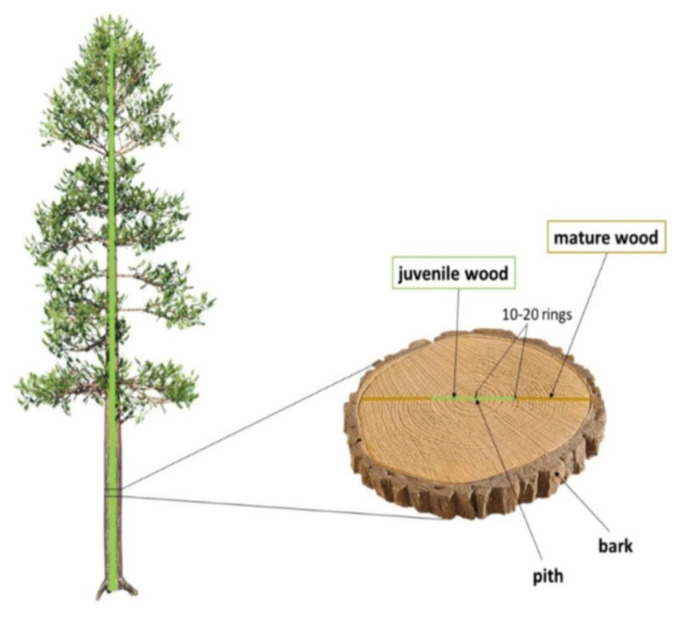
Distribution of juvenile and mature wood in the tree trunk and its cross-section. Adapted from Broda et al. [45] (open access).

Juvenile wood is a distinct type of raw material that differs from mature wood in its anatomical, physical, and mechanical properties. It forms during the early stages of tree growth and consists of the annual rings located near the pith in the cross-section of the stem. Although the number of rings classified as juvenile varies depending on the tree species and the methodology employed, it is generally defined as the first 10–20 annual rings adjacent to the pith (Figure 8) [45,46].

**Figure 9:**
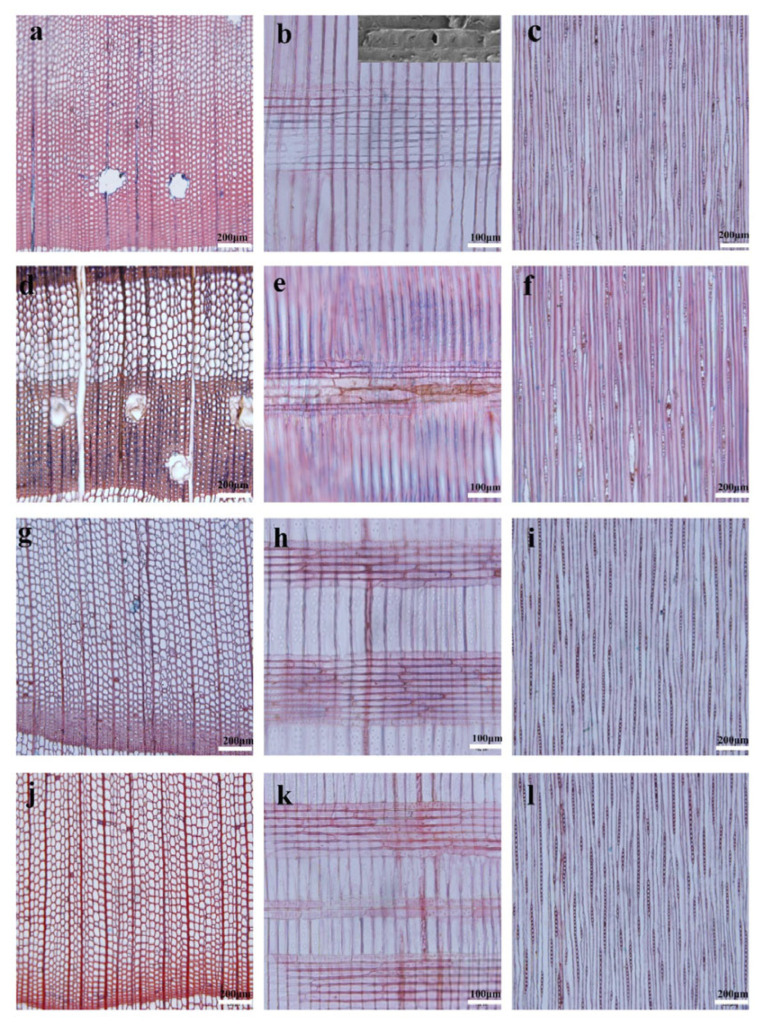
Optical micrographs of the transverse, radial, and tangential sections from Masson pine: (**a**–**c**) juvenile wood, (**d**–**f**) mature wood; Chinese fir: (**g**–**i**) juvenile wood, (**j**–**l**) mature wood. Adapted from Meng et al. [49] (open access).

Juvenile wood is characterized by considerable variability in its properties. Compared to mature wood, it generally has a lower density, higher lignin content, and lower cellulose content. Additionally, the microfibril angle in relation to the longitudinal axis of the cell is higher, and the growth rings exhibit a more pronounced spiral arrangement. Juvenile wood also features shorter, smaller-diameter cell elements with thinner cell walls [47,48]. The differences in the microscopic structure between juvenile and mature wood of Masson pine (*Pinus massonia*) and Chinese fir (*Cunninghamia lanceolata*) have been illustrated, for example, by Meng et al. [49] (Figure 9).

Such differences in anatomical structure and chemical composition result in a lower modulus of elasticity and reduced mechanical strength compared to mature wood, as well as increased susceptibility to dimensional changes in the longitudinal direction and greater deformations [46,50]. In the literature, there are varying results regarding the properties of PBs made from juvenile wood. While some studies indicate an improvement in the mechanical properties of these boards, others suggest a deterioration. This variation is attributed to the unique characteristics of juvenile wood, which depend on numerous factors including processing conditions and technological parameters. This variation in findings emphasizes the need for further research to better understand the impact of juvenile wood on the quality of the resultant boards.

**Figure 10:**
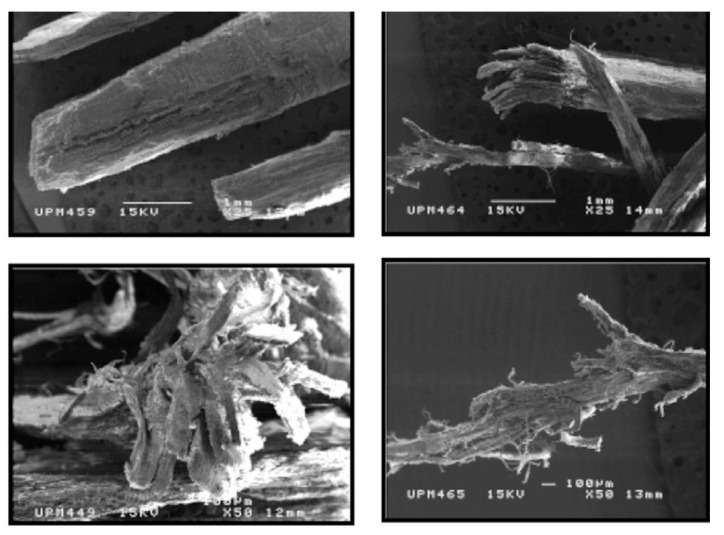
Particle geometry of rubberwood from (**a**) 25-year-old PB 260 clone; (**b**–**d**) 4-year-old RRIM 2000 series. Adapted from Paridah et al. [52] (open access).

An example of such studies is the work of Pugel et al. [47,48,51], who investigated the potential use of southern pine juvenile wood in the production of PBs, flakeboards, and fiberboards. The authors found that although the dimensional stability of the boards made from this raw material was lower compared to those made from mature wood, their internal bond strength (IB), modulus of rupture (MOR), modulus of elasticity (MOE), and durability after aging tests were at least similar, and in some cases even higher. However, it was emphasized that to achieve boards with properties comparable to those made from mature wood, higher compaction ratios should be applied, and when partially substituting juvenile wood, its proportion should be limited. Similar results were obtained by Paridah et al. [52], who used 4-year-old new clones of rubberwood from the RRIM 2000 series instead of the 25-year-old PB 260 clones typically used in most rubberwood processing plants in Malaysia. The authors demonstrated that RRIM 2002 and RRIM 2020 PBs exhibited higher MOR values, comparable to the control boards made from mature wood. In terms of IB, higher values were achieved for the RRIM 2002 and PRIM 2025 clones, although this was accompanied by an increase in thickness swelling and water absorption. The authors highlighted the distinct geometry of the juvenile wood particles and their fibrillation (Figure 10). These particles exhibited greater slenderness, which positively affected the MOR, MOE, and IB values. According to the authors, fibrillation increased the contact area between particles, enabling more efficient load transfer, contributing to the increased strength of the boards.

In contrast, Wasniewski [53] reported that although boards made from juvenile Douglas fir (*Pseudotsuga menziesii*) wood met standard requirements, with the exception of thickness swelling, their overall properties were inferior to those of boards produced from mature wood. The reduction in linear expansion of the boards associated with the increased age of the cambial wood was attributed to the decrease in the microfibril angle in the secondary cell wall of mature wood. Moreover, the significant impact of cambial age on board density was highlighted, particularly regarding the density profile. Czarnecki et al. [54], in research focusing on the potential of juvenile wood from Scots pine (*Pinus sylvestris* L.) and silver birch (*Betula pendula* Roth) in the production of PBs boards with a density of around 550 kg/m^3^, found that boards made from juvenile pine wood exhibited higher IB compared to boards made from juvenile birch or even industrial wood particles supplied from a PB production plant. The variation in strength of these boards depending on the raw material used is attributed to the increased flexibility and susceptibility to deformation of juvenile pine particles, which promote larger bonding surface areas and a thinner adhesive layer. It was noted that mature wood is less susceptible to bonding, mainly due to its higher density, thicker cell walls, and smaller pore diameters, which limit adhesive penetration into the wood structure. However, boards made from juvenile pine wood showed a 40% lower MOE, indicating reduced stiffness compared to boards made from traditional wood. Due to the higher density of birch wood, boards made with it exhibited comparable MOR and only slightly lower MOE compared to boards made from mature wood. Additionally, all boards made from juvenile wood exhibited significant thickness swelling (TS) and water absorption (WA) after 2 h of immersion in water, with the highest values observed for pine wood. The authors attributed this phenomenon to structural differences in juvenile wood, such as higher porosity (especially in birch), thinner cell walls, greater microfibril angle, and denser structure in the final boards. Similarly to the findings reported by Paridah et al. [52], the study also highlighted the distinct geometry of juvenile wood particles, particularly their greater slenderness and flatness. On the one hand, this favored better compression, improving the board structure, but on the other hand, it contributed to increased swelling, negatively affecting dimensional stability. Moreover, Muhcu et al. [55], when studying the properties of PBs made from different parts of the European larch (*Larix decidua* Mill.) trunk, found that top logs, which contain a higher proportion of juvenile wood, had lower cellulose and lignin content compared to material from lower parts of the trunk, which contained more mature wood. This resulted in reduced strength, lower compaction, and limited adhesive bonding ability. These factors, along with the higher ash content and elevated pH of juvenile wood, negatively affected the MOR and IB of the finished boards.

In the 1980s, it was recognized that juvenile wood is a determining factor in the production of OSBs, as it constitutes a significant portion of the wood’s volume [8,56,57]. This is supported by the findings of Cloutier et al. [56], who demonstrated that, similarly to PBs, the use of juvenile wood from radiata pine (*Pinus radiata* D. Don) in OSB production leads to a significant reduction in mechanical properties (MOR, MOE, and IB) and increase in swelling and linear expansion. However, the decrease in IB values can be partially minimized by using a finer fraction of strands in the core layer of the boards. Markedly, it has been shown that increasing the fine content up to 20% in the core layer of the boards results in a higher IB strength [58]. It was also found that juvenile wood can be used in the production of these boards up to 70% of the dry wood mass without significantly compromising physical and mechanical properties when it is placed in the outer layers. This aligns with the results of Pecho et al. [59], who also showed that boards containing 70% juvenile wood exhibit the best physical and mechanical properties.

**Figure 11:**
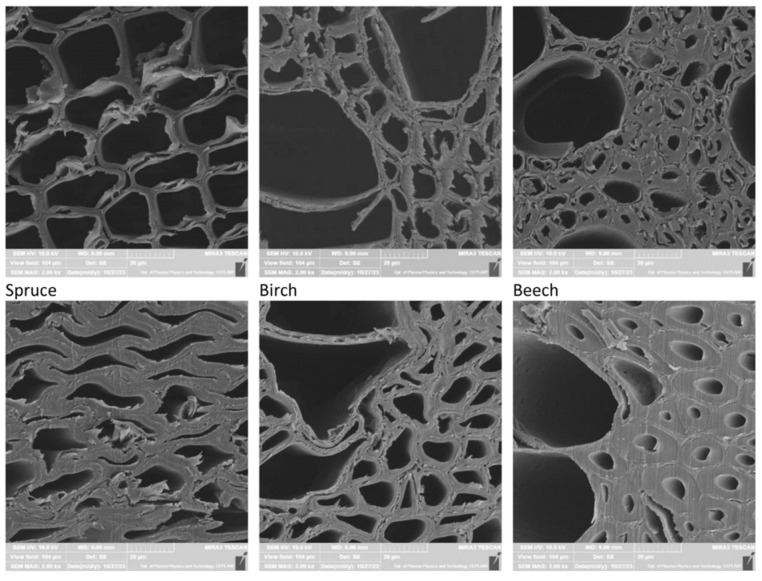
Densification of input wood and wood in OSBs. Adapted from Pipíška et al. [61] (open access).

The primary raw material for OSB production is softwood, mainly pine and spruce [60]. However, there is growing interest in using other wood species. Pipíška et al. [61] conducted studies using juvenile wood with a diameter of 100–150 mm from nine species: Scots pine (*Pinus sylvestris*), European larch (*Larix decidua*), poplar (*Populus tremula* L.), willow (*Salix alba* L.), alder (*Alnus glutinosa* L. Gaertn.), birch (*Betula pendula*), European beech (*Fagus sylvatica*), English oak (*Quercus robur*), and hornbeam (*Carpinus betulus*). The outcomes indicate that European larch, poplar, willow, and alder are promising candidates for OSB production without the need for blending with traditional softwoods, and they could serve as replacements for spruce in wood-based board manufacturing. It was found that the MOR and MOE of boards made from larch, poplar, willow, alder, and birch were comparable to those of spruce-based boards. Among these, boards made from spruce and alder exhibited the highest stiffness. No significant differences in internal bond were observed between spruce and the other tested species. The study concluded that boards made from higher-density wood demonstrated better physical properties, while those from lower-density wood (except pine) exhibited superior mechanical performance. A crucial factor influencing board properties was the compaction ratio (CR), which was higher for lower-density species, allowing all boards to reach similar final densities. As a result, OSBs made from lighter woods such as poplar or alder achieved performance levels comparable to those made from denser species. This was confirmed by SEM images (Figure 11), which showed structural changes in the wood depending on CR. In spruce with a density of 392 kg/m^3^, significant structural transformations were observed; in birch (509 kg/m^3^), cell wall deformation occurred; in beech (730 kg/m^3^), no visible differences were noted.

In summary, the use of juvenile wood in the production of PB and OSB boards represents a viable alternative to traditional raw materials. Despite certain limitations associated with the morphology and physical properties of juvenile wood, its application in board manufacturing is possible. By properly adjusting technological parameters, such as species selection, particle geometry and content, and compaction degree, it is possible to produce boards with satisfactory mechanical and physical properties. Current research suggests that juvenile wood can be successfully utilized in wood-based board production, especially at implementation levels of up to 70%, without significantly compromising the quality of the final product.

### 2.2. Wood from Fast-Growing Plantation Species

**Figure 12:**
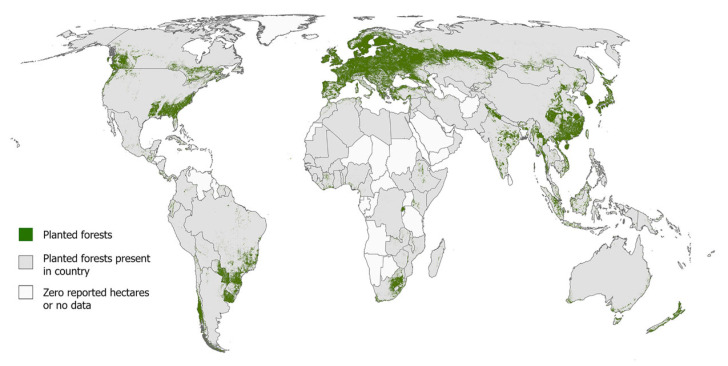
Plantation crops have the world. Adapted from Richter et al. [66] (open access).

Juvenile wood constitutes a significant component of fast-growing trees, which are typically harvested at a young age. Its proportion is much higher than in wood sourced from natural forests [62]. Due to the need to identify new sources of wood raw material, particularly for the energy sector, fast-growing tree species are gaining increasing importance. Their high availability, rapid biomass creation, and ease of processing also support the expansion of large-scale plantations [63]. The Global Forest Resources Assessment (FRA) identifies two main forest categories: naturally regenerating forests and planted forests. Natural forests cover 4.1 billion hectares, accounting for 93% of total forest area [64]. In contrast, the total area of forest plantations is estimated at 294 million hectares, or 7% of global forest area [65]. It is projected that the area of forest plantations could increase by 20 to 40 million hectares by 2050 as a means of meeting growing demand; however, their productivity depends on factors such as time since establishment, climatic conditions, the species used, and applied management practices [64]. Half of the world’s plantation forests are located in China, Russia, and the United States. Together with Canada, India, and Brazil, these countries are the largest producers of industrial roundwood from plantations globally (Figure 12) [66]. Moreover, in the past decade, the most important tree species cultivated in European plantations have been willow and poplar, with black locust and aspen playing a lesser role [67,68,69].

The properties of wood raw material obtained from plantations largely depend on its juvenile wood content, which can be effectively controlled by adjusting the rotation age. It is assumed that as the rotation age increases, the proportion of juvenile wood decreases, leading to improved mechanical properties of plantation-grown wood [70]. Fast-growing tree species are characterized by low density, a high proportion of parenchyma, and relatively high porosity, which affects their susceptibility to moisture absorption and shrinkage. These species are expected to play a major role in mitigating rising atmospheric CO_2_ levels [71]. Additionally, the price of fast-growing wood species is lower compared to that of commercial species [72]. According to estimates by Grzegorzewska et al. [73], material and energy cost savings may range from 6% to 17%. The authors reported that as early as 2014, the unit cost of materials and energy for producing boards entirely from fast-growing plantation wood could be reduced by up to USD 22.8 per m^3^. In the context of rising wood prices, such cost reductions not only represent a significant economic advantage, but also contribute to the more sustainable utilization of raw material resources.

This section focuses on selected plantation-grown tree species with high potential for use in the production of wood-based PBs, namely willow, poplar, eucalyptus, and paulownia. In the context of PB and OSB manufacturing, these species represent viable alternatives to conventional raw materials, offering favorable physical and mechanical properties while contributing to the sustainable development of the wood-based industry.

#### 2.2.1. Willow and Poplar

As previously mentioned, a significant share of fast-growing tree plantations is dedicated to the cultivation of willow (*Salix viminalis* L.) and poplar (*Populus* spp.). Both species are known for their rapid growth and suitability for energy production. They are easy to cultivate and do not require intensive care, making them cost-effective and simple to manage [74]. Additionally, willow is used in soil phytoremediation processes [75], while poplar helps stabilize soil and contributes to improved air quality due to its high carbon dioxide absorption capacity [76].

**Figure 13:**
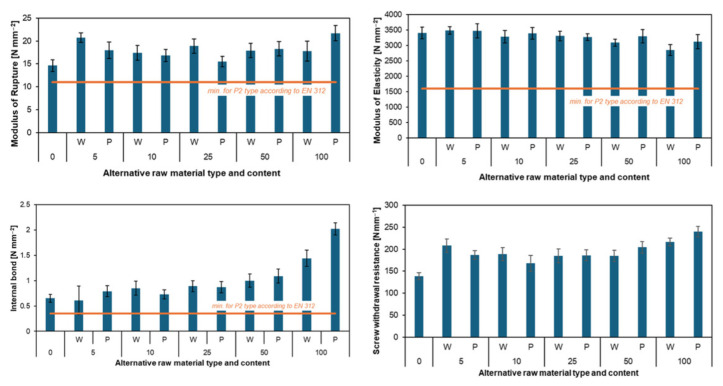
Mechanical properties of willow (W) and poplar (P) wood PBs. Adapted from Żabowski et al. [74] (open access).

**Figure 14:**
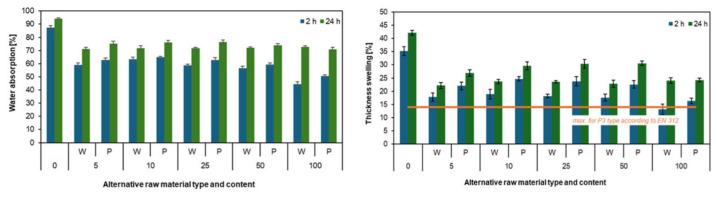
Water absorption and thickness swelling of willow (W) and poplar (P) wood PBs. Adapted from Żabowski et al. [74] (open access).

The literature presents some inconsistencies regarding the influence of these raw materials on the properties of PB, particularly in terms of mechanical strength. These differences are likely attributable to variations in bark content, moisture levels, and processing methods, which ultimately affect particle geometry and compaction [77]. Research performed by Sean and Labrecque [78] on the suitability of willow (*Salix viminalis*) for the production of three-layer PBs demonstrated that incorporating up to 30% of willow particles (in both face and core layers) generally results in better mechanical properties compared to boards made from conventional industrial wood particles. The positive effect of substituting pine particles with willow was also confirmed by Frąckowiak et al. [79], who showed that typical chipping equipment can be used to produce suitably shaped particles from various alternative raw materials. Replacing 25% of industrial pine particles in the core layer with willow particles led to higher MOR and MOE values than in control pine boards. On the other hand, Warmbier et al. [77] found that increasing the substitution rate of pine particles with willow particles slightly decreased MOR and MOE, while IB improved. However, dimensional stability, assessed by thickness swelling and water absorption, deteriorated. Different results were reported by Żabowski et al. [74], who examined boards containing various proportions of willow and poplar particles. Regardless of the substitution level, the mechanical properties of the three-layer boards (at a density of 680 kg/m^3^) not only exceeded those of the reference board but also met the EN 312 standard requirements for P2 boards (Figure 13 and Figure 14). Boards made entirely from willow and poplar exhibited higher MOR and IB values than those made from industrial wood particles, although their MOE was slightly lower. Overall, willow-based boards showed slightly lower strength than poplar-based ones but comparable dimensional stability and water absorption. However, in the case of single-layer boards, incorporating more than 50% willow wood particles results in a decrease in MOR and MOE values, but an increase in IB [80].

The suitability of poplar wood as a raw material for board production has also been confirmed by Nourbakhsh [81], who tested several three-year-old poplar clones, including *P. euramericana costanzo*, *P. euramericana 561.41*, *P. euramericana triplo*, *P. euramericana vernirubensis*, *P. euramericana marilandica*, *P. euramericana I-214*, *P. deltoides 77.51*, *P. trichocarpa*, and *P. nigra betulifolia*. Most of the manufactured boards met the strength requirements defined by EN standards (IB, MOE, and MOR), with the best results observed for the clones *P.e. costanzo*, *P.e. 561.41*, *P.e. vernirubensis*, and *P. trichocarpa*. The highest dimensional stability was recorded for *P.e. 561.41* and *I-214*. Based on these findings, it was concluded that poplar wood-based boards show potential for use in the production of furniture, wall boards, and ceiling boards. These findings are also supported by studies conducted by Kowaluk et al. [82] and Jahan-Latibari and Roohnia [83], who investigated the potential of using residues from poplar plantations.

An interesting approach was proposed by Abdulqader [22], who significantly improved board properties by mixing corn stalk particles with poplar wood particles. The resulting composite boards exhibited low water absorption and reduced thickness swelling. Moreover, tensile strength, which is typically a weak point in lignocellulosic PBs, was improved to a level suitable for use in dry conditions.

In general, both willow and poplar can be considered valuable raw materials for PB production. They can successfully compete with commercial furniture-grade PBs and, with the appropriate choice of binding agent, could also be used in structural applications, for example, in prefabricated wooden frame buildings [74].

The positive results associated with the use of willow and poplar in PBs prompt further analysis of their suitability for OSB, which, due to their primary use in construction, require raw materials of appropriate quality. According to Dumitrascu et al. [84], both willow and poplar can successfully be used in OSB production, even as individual raw materials. Boards made from poplar were found to be the most affected due to their low MOE, around 3147 N/mm^2^ (major axis), but they achieved high IB values and the highest MOR, at 43 N/mm^2^. Similar MOR values for OSBscontaining poplar wood were obtained by Akrami et al. [85]. From a practical perspective, Lunguleasa et al. [86] formulated important conclusions after comparing the properties of OSBs made from coniferous wood, commonly used in Europe (e.g., fir, spruce, and pine), with those made from deciduous wood, specifically poplar, willow, and birch. OSBs made from a mixture of deciduous wood demonstrated better mechanical properties (mainly MOE and IB) and lower thickness swelling compared to those made from coniferous wood. The authors also emphasized the significant influence of wood density on the MOE of OSB. Overall, they concluded that parameters such as MOR, MOE, IB, and WA show a good correlation with board density, whereas the relationship between TS and density is not that clear. Consequently, they recommended using deciduous wood, such as willow, poplar, and birch, in OSB production (especially in Europe) to avoid excessive exploitation of coniferous wood. This is primarily due to the favorable shape parameters and particles properties of willow and poplar.

#### 2.2.2. Paulownia

**Figure 15:**
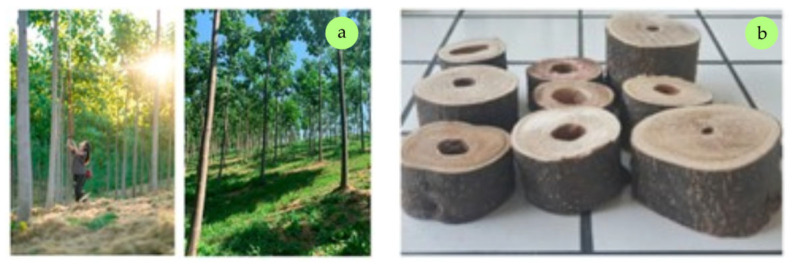
*Paulownia tomentosa*: (**a**) photo of the plantation, (**b**) cross-section with the empty pith present in the middle of the trunk. Adapted from: (**a**) Barbu et al. [92] (open access); (**b**) Esteves et al. [91] (open access).

A wood species with properties similar to willow and poplar is paulownia, belonging to the family *Paulowniaceae*, which includes ten species: *P. albiphloea*, *P. australis*, *P. catalpifolia*, *P. elongata*, *P. fargesii*, *P. fortunei*, *P. kawakamii*, *P. taiwaniana*, *and P. tomentosa*, as well as several hybrids, primarily resulting from the cross between *P. elongata* and *P. fortunei* [79,80,81,82,87,88]. Paulownia wood has a density ranging from 220 to 350 kg/m^3^, typically around 270 kg/m^3^ [89,90]. It is easy to dry and more fire-resistant than other fast-growing species due to its high ignition temperature, high moisture content, and distinctive vessel structure [91]. The wood is characterized by a high strength-to-weight ratio, low shrinkage, good processability, and good dimensional stability. Among the various species, *Paulownia tomentosa*, commonly referred to as kiri, has attracted particular interest due to its favorable physical and mechanical properties (Figure 15).

The first harvest of low-dimensional timber can be carried out after just five years [93]. The typical rotation period ranges from 12 to 15 years. The primary goal of most plantations is the production of high-value timber with diameters ranging from 40 to 60 cm. Under optimal conditions, trees can reach heights of up to 25 m and diameters between 60 and 120 cm [94]. Although paulownia wood is used in the production of plywood, paper, and everyday items, its utilization seems to remain insufficient [95]. However, studies by Nelis et al. [96,97,98] have demonstrated the potential of *Paulownia tomentosa* Spp. in the production of PBs. Their findings indicate that wood particles from paulownia can be used to manufacture lightweight and medium-density three-layer PBs, with densities ranging from 350 to 500 kg/m^3^. Notably, these boards exhibited higher mechanical properties (MOR, MOE, IB) compared to conventional boards of the same density made from industrial pine particles. The best performance was observed when paulownia particles were used in the outer layers, which enhanced the mechanical properties and reduced TS and WA. According to the authors, the improved board performance was attributed to the higher compaction of kiri particles. They emphasized the critical interaction between raw material density and the degree of compaction, highlighting that pressing intensity and particle distribution, particularly in the outer layers, had a more significant impact on board properties than the raw material alone. It was observed that incorporating as little as 33% paulownia could positively influence the mechanical performance of the boards.

In conclusion, paulownia wood can be considered not only as an alternative raw material for PB production but also as a resource capable of enhancing selected board properties, particularly in the context of lightweight and high-performance wood-based composites. This potential is further supported by findings from other researchers, who have also demonstrated the feasibility of using paulownia wood in the production of medium-density PBs [91,99,100,101]. Importantly, the performance of these boards not only meets the relevant standard requirements but in many cases exceeds them.

Although research on the use of paulownia wood in OSB production remains limited, several studies have demonstrated its potential. Salari et al. [102,103] evaluated the properties of OSBs made from *P. fortunei*, along with the possibility of enhancing their performance through the modification of urea–formaldehyde (UF) resin using nanomaterials such as nano-silica and organo-modified montmorillonite (MMT). The mechanical properties of all boards met the standardized requirements for general-purpose use in dry conditions. Furthermore, the addition of nanomaterials improved both physical and mechanical properties while reducing formaldehyde emissions. However, none of the boards met the WA and TS requirements specified for general-purpose boards. In a similar study, Wag and Chen [104] examined the effect of paulownia strands on the mechanical and orthotropic properties of OSBs. Boards containing up to 50% paulownia strands (combined with Japanese cedar or Chinese fir) showed favorable MOR and MOE values. Ultrasonic analysis revealed flake alignment levels of 93–97% in the surface layers and an orthotropy index (V-0/V-90) ranging from 2.4 to 3.2. These findings highlight the high quality of the resulting boards and support the suitability of paulownia as a raw material in OSB manufacturing.

#### 2.2.3. Eucalyptus

Another species with considerable potential in board production is eucalyptus, which is widely used in the pulp and paper industry, as well as in the manufacturing of fibreboards and solid wood products. Most eucalyptus species are rarely processed into timber due to their poor dimensional stability, frequent knots, cell collapse, high radial and tangential shrinkage, splitting, warping, and brittle heartwood during processing [65,105,106,107]. One significant challenge is the drop in the pH of eucalyptus wood and consequently the adhesive in contact with it, especially in the steam-rich environment during hot pressing of PBs, which substantially delays adhesive curing. Another drawback is the insufficient cracking of cell walls under standard board-pressing pressure, which allows full cell structure to regenerate, leading to excessive board swelling when exposed to moisture [108]. Nevertheless, compared to most softwood species, eucalyptus exhibits higher stiffness, making it well suited for structural applications. An additional advantage is its broad adaptability, as various eucalyptus species thrive in tropical, subtropical, and temperate climates.

According to estimates, eucalyptus, as one of the most important plantation-grown species, is cultivated in 95 countries worldwide, with a total plantation area exceeding 22.57 million hectares. The largest and second largest eucalyptus plantation areas are located in Brazil and China, accounting for approximately 22% and 20% of global plantations, respectively [65]. The significance of this resource is underscored by studies conducted by Da Rosa et al. [109], who investigated the use of five eucalyptus species, *E. benthamii*, *E. dunnii*, *E. grandis*, *E. saligna*, *and E. grandis*, harvested from short-rotation (7-year-old) plantations for the production of PBs. Boards manufactured from *E. grandis* exhibited the highest MOR and MOE values compared to both the other eucalyptus species and the control boards made from softwood. Meanwhile, the highest IB was observed in boards made from *E. saligna*, while the best dimensional stability, indicated by lower WA and TS, was achieved with boards based on *E. dunnii*. In general, all PBs made from eucalyptus met the requirements of European standards (EN), as well as those established by the German Standards Institute (DIN) and the Venezuelan Industrial Standards Commission (COVENIN) [110]. Further analysis by Seng Hua et al. [65] confirmed that eucalyptus-based PBs demonstrate comparable or even better bending strength compared to boards produced from other wood species. Despite their lower density, these boards often exhibit higher MOE and MOR values than those made from pine, poplar, or rubberwood. These properties are strongly influenced by particle geometry, processing conditions, and pre-treatment methods. Pan et al. [111] demonstrated that eucalyptus-based PB bonded with 4% pMDI adhesive generally exhibited better performance, except for MOR, than those bonded with UF resin. Increasing the adhesive content from 7% to 16% significantly improved board performance. Additionally, boards made from medium-sized particles (20–40 mesh) outperformed those produced from larger (40–60 mesh) or smaller (10–20 mesh) fractions in all properties except TS. Beneficial effects on board properties were also observed following hot-water treatment of the raw wood and the use of saline-treated wood. Increasing board density from 700 kg/m^3^ to approximately 840 kg/m^3^ contributed to improved dimensional stability by reducing TS after 2 and 24 h of water soaking [112]. Furthermore, studies on the biological resistance of eucalyptus-based boards have shown that the use of tannin-based adhesives and the addition of sugarcane bagasse enhance resistance to brown- and white-rot fungi and reduce susceptibility to termite attack [113].

Eucalyptus wood also represents a promising alternative raw material for OSB production. Its effectiveness has been demonstrated in studies by Iwakiri et al. [114], who produced boards with a density of 700 kg/m^3^ using six different eucalyptus species: *E. grandis*, *E. dunnii*, *E. tereticornis*, *E. saligna*, *E. citriodora*, and *E. maculata*. The results revealed a particularly high potential for *E. grandis* and *E. saligna*, as boards made from these species exhibited physical and mechanical properties comparable to or even superior to those of boards manufactured from *Pinus taeda* which is the dominant OSB raw material in Brazil. Gouveia [115] recommended blending *Pinus elliottii* strands with 75% or 50% content of *E. grandis* for OSB production. The suitability and validity of this recommendation was confirmed by Iwakiri [116], who manufactured boards with a density of 700 kg/m^3^, incorporating middle layers composed of 50% *E. grandis* and *E. dunnii*. The resulting boards met the performance requirements of Canadian and European standards and were comparable with those produced from *Pinus taeda*. Moreover, mechanical testing showed that increasing board density up to 1000 kg/m^3^ led to a significant improvement in MOE and MOR, suggesting the feasibility of using high-density OSBs for structural applications where enhanced strength is required.

#### 2.2.4. Other Plantation Species

The required parameters for PB production can be achieved using *Leucaena leucocephala*, a fast-growing, evergreen tree that often reaches heights of 7–18 m [117]. Studies on PB manufactured from *L. leucocephala*, rubberwood, and mixed tropical species in various proportions demonstrated that, regardless of the species ratio used, the boards met the minimum standards for commercial applications and were suitable for interior use in dry conditions (Type P2). Notably, the boards made from *L. leucocephala* exhibited mechanical and physical properties comparable to those produced from currently used commercial raw materials [118,119]. Rahman [117] examined the use of smaller strand sizes of *L. leucocephala* as the core layer in OSBs. The resulting OSBs fulfilled the performance criteria required for general-purpose applications, including sheathing materials for walls, floors, roofs, and other construction or renovation uses (Type OSB/1).

According to the literature, other fast-growing tree species from plantations in tropical regions can also be used in the production of both PBs and OSBs. Fabrianto et al. [120] utilized *Paraserianthes falcataria* (sengon), *Acacia mangium*, and *Maesopsis eminii* for the production of OSB/1. It was shown that boards produced from a combination of high-density and low-density species exhibited better dimensional stability than those made from a single species. Moreover, boards made from *Neolamarckia cadamba* (kelempayan), cultivated in Malaysia, have shown potential for furniture applications [121]. Yunianti et al. [122] investigated PBs produced from jabon wood (*Anthocephalus cadamba*), commonly grown in Southeast Asia (e.g., Indonesia, Malaysia, India). By modifying the wood using a combination of hydrogen peroxide (H_2_O_2_) and ferrous sulfate (FeSO_4_), binderless PBs were produced with physical and mechanical properties that complied with the relevant standards.

In the wood industry, particularly in North America, softwood raw materials from plantations are already being processed. The most commonly cultivated species include Loblolly pine (*Pinus taeda*), Ponderosa pine (*Pinus ponderosa*), and Sitka spruce (*Picea sitchensis*). However, there are species with significant potential that are not yet widely utilized in the wood-based board industry. One such example is European larch (*Larix decidua*), which, despite being cultivated on plantations, has not yet found broader application in wood-based materials production. Nevertheless, there is growing interest in the scientific literature regarding its potential as an alternative wood resource. Larch (primarily *Larix decidua*, as well as *Larix kaempferi* and their hybrid *Larix* × *eurolepis*) is cultivated on a smaller scale compared to species such as poplar, eucalyptus, or paulownia. It is considered promising due to its relatively high natural durability, fast growth, and strong adaptability [123]. According to Reh et al. [124], this species is considered a promising candidate for the core layer of PBs due to its similarity to spruce, which remains one of the primary wood species used in PB production. This was confirmed by Pazio and Boruszewski [125], who produced single-layer particle-fibrous boards from plantation-grown European larch. Boards containing at least 50% fibers exhibited MOR and MOE values comparable to those of pine-based materials and showed significantly lower TS than boards made from forest-grown wood. Muhcu et al. [55] investigated the effect of log position within the tree stem on the physical, mechanical, and surface properties of PBs made from *L. decidua*. Logs were divided into five segments from the butt to the top of the tree. Results showed that with increasing tree height, fiber wall thickness and length decreased, while lumen diameter also declined. Additionally, lignin and cellulose contents decreased while hemicellulose content increased. The highest solubility values (in cold and hot water, alcohol–benzene, and sodium hydroxide) and pH were observed in the butt log. Increasing log height negatively affected mechanical properties (IB, MOR, and MOE), physical properties (WA and TS), and surface quality (wettability and surface roughness). The boards with the best properties were produced from wood located 0–3 m above ground. A valuable complement to these findings is the study by Bardak et al. [126], who analyzed larch-based PBs in terms of the effects of heartwood and sapwood content on physical, mechanical, and surface properties, as well as formaldehyde emission. Boards made entirely from sapwood had the smoothest surface and lowest contact angle. On the other hand, boards made entirely from heartwood exhibited the roughest surface and highest contact angle. Additionally, heartwood boards showed the lowest formaldehyde emission and the lowest thickness swelling compared to those made from whole wood or sapwood. The highest mechanical strength (IB, MOR, and MOE) was found in boards made from sapwood, followed by those made from whole wood, and the lowest values were recorded in heartwood-based boards. These results indicate that sapwood boards exhibit better wettability and smoother surfaces than those made from whole or heartwood material.

### 2.3. Alternative Non-Plantation Wood Species

In the search for alternative raw materials for the board industry, particular attention is still given to wood sourced from natural forests rather than plantations. Species such as birch, beech, and alder exhibit relatively fast growth rates, making them potential candidates for the production of various wood-based materials, including PBs and OSBs. Their rapid growth and widespread availability in many regions enhance their attractiveness as substitutes for more conventional species. This section discusses the potential of these native hardwoods and tropical fast-growing non-plantation species for wood-based materials manufacturing, focusing on their physical and mechanical properties and the feasibility of integrating them into current production technologies while maintaining required quality standards.

#### 2.3.1. Native Hardwood Species

**Figure 16:**
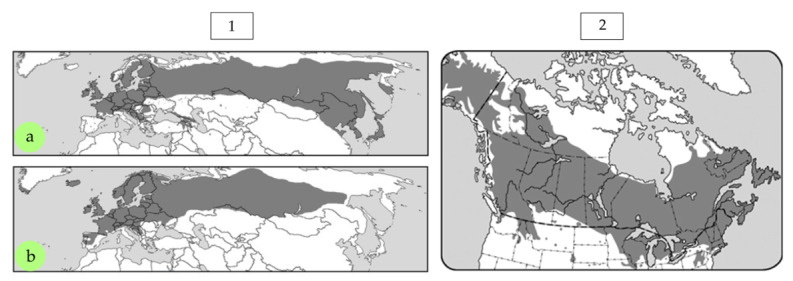
The area of occurrence of birch in Eurasia and North America: 1—Eurasia: (**a**) silver birch and (**b**) white birch; 2—white birch in North America. Adapted from: 1—Dubois et al. [127] (open access); 2—Theriault et al. [128] (open access).

Birch is distributed across much of North America and throughout Eurasia (Figure 16), and it has the widest range of all European deciduous species [127]. In Northern Europe, birch plays a key role in plywood production, which has led to extensive research interest in the use of birch wood and bark for wood-based board manufacturing [124]. Despite its significance and availability, relatively few studies have focused on the use of birch wood alone in the production of PBs and OSBs. The potential of birch wood for manufacturing low-density PBs has already been demonstrated in previously discussed work on juvenile wood [54]. Birch-based boards showed higher bending strength and better dimensional stability compared to those made from industrial wood particles.

In the case of OSBs, Brunette [129] demonstrated that the IB of white birch boards is higher than that of aspen and balsam poplar, although their modulus of elasticity and bending strength are generally inferior. Additionally, the good surface quality of birch contributes to more effective resin distribution and improved bonding quality. According to the author, producing boards with a density of 640 kg/m^3^ and 60% birch content meets the normative requirements for both strength (MOR, MOE, and IB) and dimensional stability. It was generally concluded that MOE is a crucial property that limits the utilization of white birch in OSBs. This finding is supported by Beck et al. [130]. The hypothesis was also raised that particle thickness should be adjusted according to wood density to achieve boards with the desired mechanical properties. According to Brunette [129], using particles with a thickness of 0.64 mm yields the best parameters for boards made from white birch in the core layer and balsam poplar in the outer layers. This was confirmed by Yong [131] who showed that using thinner particles (0.51 mm) in the core layer of trembling aspen boards results in comparable IB values to commercial aspen boards; however, increasing their thickness leads to a deterioration in IB strength without affecting MOR and MOE. On the contrary, Dumitrascu et al. [84] concluded that alongside willow and poplar, birch wood can also be effectively used in OSB production, achieving similarly good results. Among the species analyzed, birch boards demonstrated the best performance in terms of MOR and MOE, with only slightly lower IB. Despite the noted differences in bending strength and stiffness values, it is important to note that, regardless of the manufacturing conditions, all boards met the minimum normative requirements. The feasibility of using birch wood was also discussed in the context of fast-growing plantation wood for OSB production, particularly in a mixture with willow and poplar, as presented by Lunguleasa et al. [86].

**Figure 17:**
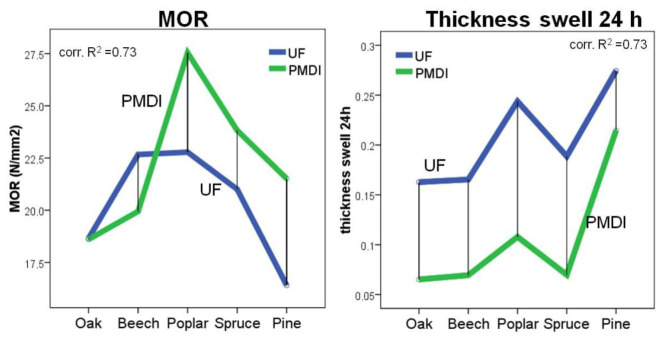
Bending strength and thickness swelling of PBs depending on the species of wood from which the particles were obtained and the type of bonding agent. Adapted from Wimmer et al. [135] (open access).

Another species that has attracted the interest of researchers is beech, which Wimmer et al. [132] considered an “allrounder.” European beech (*Fagus sylvatica* L.) is one of the most important and widespread tree species in Europe, primarily distributed in Central and Western Europe [133]. According to Iždinský et al. [134], due to its availability and potential for cost reduction, beech wood (*F. sylvatica* L.) can also be used in the production of PBs. It was shown that introducing specific amounts of beech particles significantly improved the properties of PB made from spruce wood and 20% recycled wood. An improvement in dimensional stability was achieved, although there was a slight reduction in their MOR strength. Beech particles had a positive effect on IB values and surface soundness. With 20% and 30% beech particles, the PBs met the requirements for board type P2. In the study performed by Wimmer et al. [132], it was shown that PBs made from beech wood performed well in terms of most properties. These parameters were also compared with those of boards made from spruce, pine, poplar, and oak. It was found that oak had the lowest results in terms of TS and IB, while poplar exhibited better bending properties. Clearly, in order to achieve the required quality of the boards, not only the wood species from which the particles were sourced is important, but also the strong interactions with the resin type and the applied primer treatment [132]. This is confirmed by the data shown in Figure 17, which illustrates the relationship between bending strength, swelling, and the wood species and binding agent type.

In addition to the previously discussed wood species, fruit trees can also be utilized in the production of PBs. This type of raw material is currently undervalued by the wood industry, despite estimates indicating that its resources amount to 0.35 m^3^/ha [136]. Most of the wood harvested from orchards is currently burned. For example, Kowaluk et al. [137] used biomass from apple (*Malus domestica* Borkh.) and plum (*Prunus domestica* L.) tree pruning. When producing three-layer PBs, it was shown that the particles from apple wood had significantly higher bulk density compared to the traditional pine raw material—over 40% higher in the outer layer and 49% higher in the middle layer. Compared to the reference boards, the apple wood boards exhibited higher IB and screw withdrawal resistance (SWR), but lower MOR and MOE values. Additionally, these boards showed lower swelling and water absorption after 24 h of immersion. On the other hand, the boards made from plum wood demonstrated higher IB values, while other properties were slightly lower. Nevertheless, the properties of PBs made from pruning waste of fruit trees meet, and even exceed, the minimum requirements of European standards. This corresponds with the findings of Lykidis et al. [138], who demonstrated that replacing coniferous wood particles with fruit wood particles in proportions above 50% improved the IB of the boards, regardless of their density.

#### 2.3.2. Tropical Fast-Growing Species

The species discussed earlier, such as birch, beech, and fruit tree wood, are widely used raw materials in temperate climates. In tropical regions, however, attention is drawn to other non-plantation tree species that could serve as alternative sources of raw material as well. Tropical regions, particularly South America, show great potential for using alternative raw material sources in the production of wood-based boards from non-plantation areas. One example is *Erisma uncinatum*. Ferro et al. [139] compared the properties of *Erisma uncinatum* boards with *Schizolobium amazonicum* and pine boards, all bonded with polyurethane adhesive. They confirmed the feasibility of producing OSBs from *Erisma uncinatum* wood, which exhibited the best physical properties (lower water absorption) due to its higher density and lower porosity compared to the other species. The best mechanical properties were achieved in the case of pine boards. In the context of sourcing raw materials, other Amazonian species such as *Byrsonima crispa*, *Eschweilera coriacea*, *Eschweilera odora*, *Manilkara amazonica*, *Pouteria guianensis*, and *Swartzia recurva* have also been investigated [140]. Based on a comparative analysis with the requirements of European standard and a reference board made from *Pinus Taeda*, it was found that species like *Byrsonima crispa*, *Eschweilera coriacea*, *Eschweilera odora*, and *Pouteria guianensis* have great potential for PB production. However, species like *Manilkara amazonica* and *Swartzia recurva*, despite their high density, did not provide sufficient bending strength and stiffness for PB boards.

Aguiar et al. [141] assessed the properties of OSBs produced from the following Amazonian species: *Caryocar villosum* Aubl., *Erisma uncinatum* Warm., and *Hymenolobium excelsum* Ducke. Based on the outcomes, it was found that the wood-mixed boards achieved higher mechanical strength due to the use of wood from tropical species instead of pine wood. According to the authors, a mixture of these three species, in addition to pine, could be used to introduce a new competitive product to the traditional OSB market.

In summary, it should be stated that although Amazonian species show potential for use in the production of PBs and OSBs, their application should be closely linked to the principles of sustainable forest management. In the face of ongoing Amazonian degradation, it is crucial to seek solutions that combine the development of the wood industry with the protection of biodiversity and the integrity of forest ecosystems. Nevertheless, as noted by Iwakiri et al. [142], the use of residues from primary and secondary processing of tropical wood and the study of these species in the context of using them as raw materials in boards production can provide support for the installation of PB and OSB factories in the Amazon region.

### 2.4. Recycled Wood

**Figure 18:**
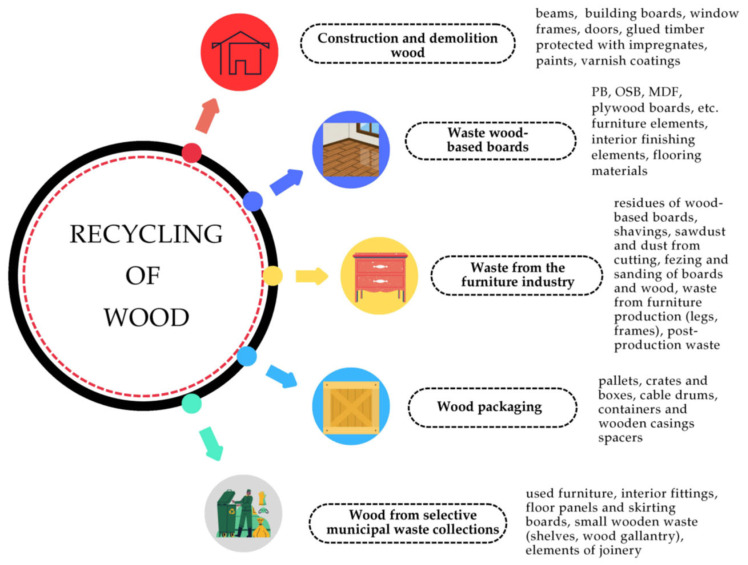
Main sources of waste wood for of recycled wood for application in the wood-based board industry.

The growing demand for wood-based materials has intensified the pressure on primary forest resources. In response, the incorporation of recycled wood into the production of OSBs and PBs has emerged as a sustainable alternative. Recycled wood offers a valuable source of raw material that aligns with circular economy principles by extending the life cycle of wood products [143]. Its use contributes to resource efficiency by minimizing logging activities and reducing the dependency on virgin timber [144]. Furthermore, incorporating recycled wood helps divert substantial volumes of post-consumer wood waste from landfills and energy recovery facilities, thereby conserving landfill space and mitigating greenhouse gas emissions associated with wood decomposition and combustion. The availability of recycled wood in various regions makes it an accessible and cost-effective feedstock for the wood-based materials industry. Its inclusion in board manufacturing supports the environmental objectives outlined in the European Green Deal. Moreover, utilizing recycled wood can help meet legislative targets for waste reduction and renewable material use. While variability in its physical and chemical composition poses certain processing challenges [145,146], advancements in sorting and pre-treatment technologies continue to improve its viability. The substitution of virgin raw materials with recycled wood may also mitigate market volatility in timber prices. From an industrial perspective, integrating recycled wood into OSB and PB production may foster innovation in adhesive systems and board design. It also encourages the development of grading and certification systems to ensure safe and efficient utilization. Finally, promoting the use of recycled wood supports the sustainable green and digital transition of the wood-based board industry. An example classification of recycled wood based on its source is shown in Figure 18. This section focuses on recycled wood derived from construction and demolition, furniture, and packaging, as these are identified by Nguyen et al. [147] as the most common sources of waste wood streams.

#### 2.4.1. Recycled Wood from Construction and Demolition

**Figure 19:**
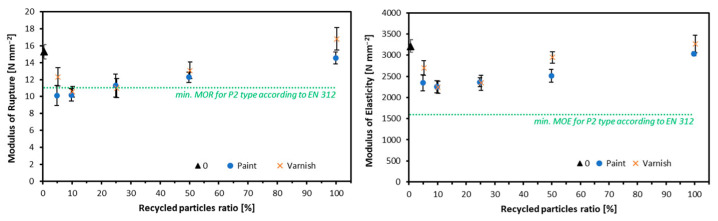
The effect of recycled particles on mechanical properties of PBs. Adapted from Wronka and Kowaluk [150] (open access).

The construction and demolition sector is the largest source of wood waste in Europe, where wood accounts for 20–40% of total construction and demolition waste, including materials from construction, rebuilding, and demolition activities [143]. An investigation performed by Azambuja et al. [148] examined the suitability of wood residues sourced from construction and demolition waste, specifically medium-density PBs (MDP), plywood, and timber, for partial substitution of industrial pine particles in the production of three-layer PB, focusing on their application in the core layer. The materials were manually segregated by type, processed into particles, and incorporated at 25% and 50% replacement levels. A range of physical (WA and TS) and mechanical (MOR, MOE, IB, SCR) properties was evaluated, along with non-destructive testing using a stress wave timer. Results indicated that all recycled wood types met IB strength requirements, confirming effective adhesion with virgin pine particles. Notably, the incorporation of 25% MDP, plywood, or timber residues did not adversely affect any measured physical or mechanical properties. In contrast, mixed, non-segregated residues showed more variable performance, highlighting the importance of source-specific separation. Additionally, the inclusion of recycled material improved the predictive accuracy of MOE via non-destructive testing methods. These findings demonstrate that up to 25% of properly segregated demolition wood residues can be reliably used in PB manufacturing without compromising product quality, contributing to more sustainable material cycles in wood-based industries. A complementary study was also performed by Azambuja et al. [149], further exploring the use of wood residues from construction and demolition sources obtained from a recycling facility. These materials were processed into particles and used to produce PBs bonded with UF resin at a target density of 0.75 g/cm^3^. When compared to reference samples made from industrial *Pinus* spp. particles, the products containing recycled timber exhibited the most favorable mechanical performance, with IB strength values comparable to the control. Among the tested residues, timber also holds practical significance due to its higher representation in collected construction and demolition waste streams. All residue types, MDP, plywood, timber, and an equal-part mixture, demonstrated acceptable IB, suggesting their potential as components for the inner layer of MDP products. However, reductions in MOR and MOE values in static bending were observed, indicating that further refinement of processing variables may be necessary to improve bending properties. These findings reinforce the viability of using demolition wood in PB production while emphasizing the need for targeted adjustments to enhance structural performance. Another study by Wronka and Kowaluk [150] addressed the suitability of using recycled wooden window frames, coated with either clear varnish or white paint, as a raw material for PB production. Window frames were seasoned, shredded, and incorporated into three-layer boards at varying proportions (0–100 parts by weight). Results showed that increasing the share of recycled wood improved MOR and MOE, particularly at substitution levels above 50 parts by weight, although not all variants met the MOR standard requirements (Figure 19).

The IB remained a limiting factor across all formulations, suggesting that improvements in bonding techniques or raw material preparation are needed to ensure structural integrity. Interestingly, the type of coating played a crucial role: clear varnish enhanced dimensional stability, reduced thickness swelling and water absorption, whereas white paint contributed to a higher proportion of fines, increasing density but potentially compromising strength properties. Markedly, both formaldehyde and total volatile organic compound (TVOC) emissions from the recycled variants remained within acceptable levels, attributed to the relatively recent coatings and extended seasoning time. The findings demonstrate that window joinery waste can be repurposed effectively into PBs, provided production processes are optimized according to the specific characteristics of the recycled input. A review by Gayda [151], focused on the Ukrainian context, highlighted the growing strategic importance of post-consumer wood, also derived from wooden construction components, for PB manufacturing. The increasing costs of virgin raw materials, stringent waste disposal regulations, and competitive market pressures have driven the substitution of traditional wood with recycled post-consumer wood (PCW), offering potential raw material cost reductions of 40–70%. Notably, recycled wood typically has a lower moisture content (~20%) than virgin wood, resulting in reduced drying energy requirements and improved processing efficiency. Advances in grinding and contaminant removal technologies, including over-band magnets, eddy current separators, and trammel screens, have made large-scale recycling both technically and economically viable. The resulting boards exhibit favorable mechanical properties and decreased resin and raw wood consumption. However, the review also emphasizes the need for clear regulatory guidance, particularly regarding the handling of treated wood waste and mixed PCW streams. While clean PCW poses low environmental or health risks when quality controls are implemented, treated materials remain subject to waste regulations unless further evidence justifies their safe reuse. The study calls for further development of quality protocols and contaminant removal technologies to ensure the sustainable and safe incorporation of PCW into wood-based product manufacturing in Ukraine. These insights support the broader trend toward circular economy practices in the PB sector and underline the role of PCW in addressing both material shortages and environmental goals.

#### 2.4.2. Recycled Wood from Furniture and Packing Materials

**Figure 20:**
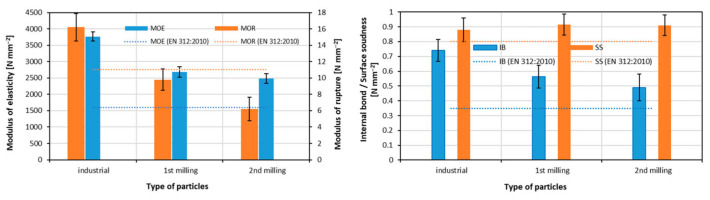
Properties of PBs manufactured from multiple recycled boards. Adapted from Wronka and Kowaluk [160] (open access).

The recycling of furniture has become an established practice in many developed countries, beginning in the 1990s. Nations such as the United States, Germany, and the United Kingdom have made significant progress in recycling waste wood from furniture, incorporating it into the production of wood-based boards such as PBs. Countries like Japan and Australia have also developed efficient systems, using recovered wood for various purposes, including board manufacturing. Germany, for example, has implemented strict regulations for managing waste wood, ensuring its effective recycling and reuse in diverse industries, including wood-based production [152,153,154]. The study performed by Lykidis and Grigoriou [155] investigated the potential of using recycled wood from laboratory PBs in the production of new boards through four different hydrothermal treatments. The results indicated that recycled boards showed a decrease in most mechanical properties compared to the control boards, with the exception of the MOE. The second recycling process caused a more pronounced deterioration in the properties, particularly affecting hygroscopic characteristics and reducing formaldehyde content. Among the hydrothermal treatments tested, the 6 bar/156 °C/45 min treatment resulted in the most significant quality degradation of the recycled boards. These findings highlight the importance of optimizing the recycling process to minimize quality loss, with milder conditions showing potential for better preservation of the mechanical properties of recycled PBs. A study of Luo et al. [156] confirmed that recycled PBs can serve as a viable raw material in new PB production and showed that the cured UF resin can be effectively decomposed to recover usable wood particles. In this study, liquid hot water (LHW) pretreatment was applied to break down UF resin and release wood particles from used PBs. These recovered particles were then blended with fresh industrial particles at varying ratios. Although the recycled boards exhibited lower mechanical properties, mainly MOR and MOE and IB, compared to boards made exclusively from virgin particles, their 2 h TS was improved. The PBs containing up to 40% recycled particles still fulfilled the Chinese National Standard requirements for general-purpose applications, confirming the suitability of partial substitution without compromising the functional properties. The research conducted by Iždinský et al. [157] investigated the effects of incorporating recycled wood particles from various sources into UF-bonded PB. The results showed that PBs containing recycled particles from waste PBs exhibited significantly improved moisture properties, with reductions in TS and WA by up to 59% and 51%, respectively. However, mechanical properties such as MOR, MOE, and IB decreased as the proportion of recycled material increased, particularly in boards made entirely from wastes. The biological resistance of the PBs varied depending on the type of recycled material used. The study concluded that PBs with recycled wood particles can be suitable for applications less exposed to stresses, but the type and amount of waste wood used should be carefully considered to ensure compliance with quality standards. Furthermore, another study showed that PBs with some of the recycled materials had faster burning rates and shorter ignition times compared to those made from sound spruce wood. Additives in recycled materials also reduced fire resistance, increasing mass loss. However, PBs made from minimally processed solid wood, like spruce logs and pallet boards, showed improved fire resistance, with longer ignition times and delayed peak burning rates. The results emphasize the importance of material selection and processing in optimizing PB fire safety, suggesting future research focus on balancing fire safety, sustainability, and cost-efficiency [158]. Study of Czarnecki et al. [159] investigating the incorporation of recycled wood-based materials into PB production have shown promising results, particularly when such materials are used in the core layer. Experimental PBs were manufactured by replacing up to 60% of the core-layer wood particles with waste derived from raw and laminated UF-bonded PBs, phenol-formaldehyde (PF)-bonded water-resistant boards, and MDF. When up to 50% of UF-bonded recycled board particles or laminated board particles were used, there was no significant reduction in the physical or mechanical properties of the final product. Moreover, PF-bonded board particles could be added at rates up to 60% without deterioration, provided the boards were glued with the same PF resin. The most favorable outcomes were achieved using waste MDF, where even a 60% addition slightly reduced the MOR but improved IB and TS. Importantly, none of the recycled materials used negatively impacted the safety or health standards of the final PBs. The study by Wronka and Kowaluk [160] addressed the challenges of multiple mechanical recycling cycles. Re-milling PBs, already composed of non-wood additives like thermoset resins and laminates, led to a significant reduction in particle size and unfavorable changes in particle morphology (increased fine fraction and shortened shapes). Boards produced entirely from second-milling particles exhibited significantly reduced mechanical properties, particularly in MOR and IB (Figure 20). However, physical properties such as TS and WA improved, likely due to the accumulation and partial hydrolysis of cured UF resin. Despite the diminished mechanical performance, surface soundness remained acceptable. Additionally, a slight increase in formaldehyde and TVOC emissions was observed. While full substitution with re-milled material is not viable for structural applications, such boards may be suitable for non-load-bearing uses, like door cores or acoustic insulation. However, authors noted that further research is needed to define the optimal proportion of recycled particles that balances sustainability with performance.

According to Zhang et al. [152], the reuse of recycled wood from furniture offers both environmental and economic advantages in the production of PBs. Moreover, according to authors, material characteristics, processing methods, and product requirements should be carefully considered. To further improve the efficiency and quality of recycling processes, structured evaluation models, such as those incorporating fuzzy hierarchical analysis and Technique for Order Preference by Similarity to Ideal Solution (TOPSIS), can facilitate informed decision-making by classifying recyclability and optimizing material flow. Integrating such tools into the PB production chain could enhance the selection of suitable waste inputs, optimize processing strategies, and contribute to the establishment of standardized systems for assessing and utilizing recycled wood. Continued refinement of these evaluation frameworks is essential to address material variability and to support a circular approach within the wood-based material industry.

**Figure 21:**
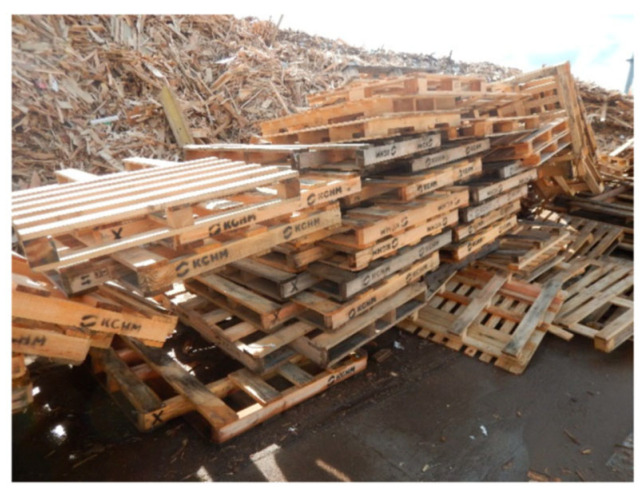
Waste pallets used for PB production. Adapted from Iždinský et al. [162] (open access).

In addition to wood-based boards used in furniture and packaging production, other packaging and transport materials, such as wooden pallets, were also examined for their potential use in manufacturing PBs from recycled wood. The pallet market is expanding due to improvements in goods transportation standards, increased use of modern material handling systems, and growing demand for palletized goods across industries. By 2018, the global pallet market reached an estimated 6.87 billion units, with over 600 million standardized wooden pallets in circulation and 123 million produced in 2019 which is an increase of 1.2 million compared to the previous year [161]. A recent study of Iždinský et al. [162] examined the potential of using recycled spruce pallets (Figure 21) as a raw material for PB production, comparing their performance with PBs made from fresh spruce logs.

The results showed that the type of spruce particles used had no significant influence on water-related properties such as TS and WA. However, a notable reduction in mechanical properties was observed in PB made from recycled spruce pallets. Specifically, the MOR decreased by up to 31.5%, the MOE by up to 23.1%, and the IB by up to 22.8%, indicating weaker structural performance. Additionally, recycled particles reduced decay resistance against the brown-rot fungus *Serpula lacrymans* by up to 15.4%, although mold resistance remained unaffected. These deteriorations in performance were attributed to the presence of undetected additives such as biocides or paints and biological degradation from fungi or insects in the recycled wood, which negatively impacted adhesion and bond quality. Despite these limitations, the study highlights the environmental and economic benefits of incorporating recycled wood into PB production and emphasizes the need for improved sorting and cleaning technologies. The authors recommend further research using various recycled wood sources and their combinations to better understand and optimize board properties.

## 3. Prospects and Challenges of Using Alternative Wood Raw Materials in PB and OSB Production

The production of PB and OSB faces the challenge of ensuring a stable and sustainable raw material economy. As a result, there is growing interest in utilizing less conventional wood sources, such as fast-growing species from plantations in temperate and tropical zones as well as raw materials derived from recycling and wood industry waste. This broad range of wood assortments offers potential for increasing raw material availability and diversifying products in terms of their physical–mechanical properties and applications. Low-density species, such as, for example poplar, paulownia, can be used in lightweight constructions and as furniture components, while higher-density wood, such as larch, can positively affect the strength and durability of boards used in construction. Table 1 summarizes the main benefits of utilizing alternative wood raw materials. The most significant advantages include their easy availability, often due to the possibility of plantation cultivation and short rotation cycles, which translates to more predictable supply and more efficient production planning. Another undeniable advantage is the lower cultivation costs, which can directly reduce the cost of manufacturing finished boards. An additional benefit is the reduced pressure on natural forest resources by limiting the extraction of wood from ecosystems with high environmental value.

Despite these promising prospects, there are several challenges associated with the practical implementation of alternative raw materials. As noted by Neitzel et al. [163], the industrial production of wood-based boards is a process that has been optimized over decades, and modifying the handling and processing of raw materials requires long-term optimization efforts. Therefore, the adoption of non-standard raw materials can pose a significant challenge for the wood-based board industry. Key challenges include the diversity of species and their physical and mechanical properties, which complicates the standardization of the production process and the properties of the final boards. This is supported by the comparison of PB and OSB parameters, prepared based on published studies, presented in Table 2. It should be emphasized that factors such as the size and geometry of wood particles, fractional composition and proportion of the specific fraction, as well as technological conditions of production, including raw material moisture content, pressing parameters, type and amount of adhesive used, also have a significant impact on the quality of boards. These parameters are crucial for obtaining the appropriate physical and mechanical properties of the boards, and their optimization in the case of alternative raw materials requires additional research and adaptation of the production technology.

It appears that there may be practical limitations to the proportion of traditional raw materials that can be replaced. These limitations result not only from the physical and chemical properties of alternative raw materials, such as differences in density, dimensional stability, or binding performance, but also from technological aspects, such as the compatibility of raw material processing with board production processes. Factors such as variability in raw material quality, impact on the mechanical properties and durability of the final product, and economic and logistical issues, including supply stability and procurement costs, are also important. Furthermore, the lack of a clear classification of such raw materials and the failure to standardize procedures for assessing their suitability make it difficult to evaluate them, which translates into limitations in their use. This view is also supported by Reh et al. [124], who suggest that not all wood species can serve as a complete replacement for traditional raw materials given that their occurrence in forests and wood reserves is relatively low, although they can reduce the amount of coniferous raw material used in PB and OSB production. For example, research by Cloutier et al. [56] suggests the use of juvenile wood in amounts up to 70%, while Wag and Chen [104] recommend a 50% share of paulownia wood in OSB production.

Another issue is the varying chemical composition of these species. For instance, the wood of tropical fast-growing species generally contains high levels of tannins, resins, extractives, and even essential oils, which can hinder the penetration of adhesive resin into the wood cellular structure, slowing the curing process and ultimately weakening the durability and strength of the adhesive bond. Consequently, the quality of bonding is poor, leading to a lower IB. Another potential issue is the variability in the pH of different wood species, which may weaken the internal bond. In such cases, the use of adhesives with higher tolerance for the variable chemical properties of wood, such as polymeric methylene diphenyl diisocyanate (pMDI) adhesives, may be necessary. While effective, these adhesives are significantly more expensive than traditional formaldehyde-based resins [164]. Such a problem was observed by researchers in the case of eucalyptus [108]. Therefore, it should be noted that using such a wide range of raw materials will require manufacturers to adopt a customized approach, not only in selecting the type and amount of binding agent, but also in the pressing conditions.

The use of waste and recycled raw materials aligns with the principles of a circular economy and strategies aimed at reducing CO_2_ emissions. One of the main challenges is the contamination of materials, especially furniture sourced from selective municipal waste collection, which may contain coatings, laminates, various adhesives, metals, and other additives. Moreover, this type of raw material is primarily used in the production of PB, as OSB requires material that allows for the production of particles with the proper geometry. Recycled wood comes from various sources, for example, the furniture industry, transport packaging, construction, and households, which means variability in structure, density, degree of processing, and content of additional substances. Such diversity requires precise sorting and pre-treatment, which increases costs and prolongs the preparation time for raw materials in production. This heterogeneity in recycled material poses significant technological barrier. Waste from the sawmill industry, such as wood shavings and chips, is characterized by high uniformity and purity, making it well suited for use as a feedstock in PB production. In contrast, wood from wooden packaging, after the removal of contaminants and metal elements, constitutes a valuable raw material for shredding without the need for more advanced preparatory treatments. Looking towards the future development of the wood-based board industry, the role of recycled wood will continue to grow in importance. However, a necessary condition for this is the development of efficient systems for the collection, sorting, and processing of wood waste, as well as investments in technologies that enable effective cleaning and preparation for reuse. In the long term, wood recycling not only contributes to the conservation of natural resources, but also meets the growing market demand for sustainable and eco-friendly production.

In summary, the rational and strategic utilization of a diverse range of raw materials—including exotic tropical species, fast-growing plantation wood, and post-consumer or industrial wood waste—can significantly enhance the efficiency, adaptability, and sustainability of wood-based material production. This approach not only broadens the resource base but also supports circular economy principles by reducing dependence on virgin forest resources.

For this reason, further research is necessary, which should focus on optimizing the processing conditions for unconventional raw materials with various physical and chemical properties, improving adhesive systems that are resistant to contamination and raw material variability. Another important area is the analysis of the long-term impact of alternative raw materials on the mechanical properties and durability of boards, the development of methods for assessing their quality, and the creation of classification procedures enabling their wider industrial application. It is also worth conducting research on the impact of chemical and physical modification of raw materials on their technological suitability, the possibilities of combining different types of biomass, the assessment of the life cycle of products based on substitute raw materials, and the development of predictive models supporting the selection of appropriate raw materials for specific applications. This research should be supplemented by analyses of profitability, logistical availability, and the potential for integrating new material streams into existing production infrastructure, as well as an assessment of their cost effectiveness under actual industrial conditions. Furthermore, it is essential to establish robust market incentives and regulatory frameworks that encourage innovation and support the integration of alternative raw materials into mainstream production practices. These combined efforts will be pivotal in advancing the sustainable transformation of the wood-based materials industry.

## Figures and Tables

**Table 1:** Advantages and disadvantages of alternative wood raw materials compared to traditional wood raw materials.

Type of Raw Material	Advantages	Disadvantages
Juvenile wood	readily available and rapidly renewable low cost lower density may reduce energy consumption during pressing easier to compress, improving compaction and board structure good bonding potential due to greater particle contact area lower lignin content facilitates processing good potential for effective bonding in lower-density boards potential for producing lightweight boards	high variability in properties due to species and growing conditions anatomical differences between juvenile and mature wood reduced dimensional stability need for optimized production conditions due to variability in properties flexibility of juvenile wood particles can lead to greater deformation requires careful control of particle geometry higher lignin, hemicellulose, and ash content in some cases, reduced strength compared to mature wood
Fast-growing plantation species	fast growth, high yield in short periods low-cost, abundant raw material for regular harvesting easy to process suitable for lightweight boards some species can provide good strength-to-weight ratios suitable for non-structural application and interior use reduce pressure on natural forests suitable for large-scale production	quality can be inconsistent due to rapid growth limited availability of large-sized roundwood lower durability, often requiring chemical treatments limited dimensional stability of some species typically lower strength and mechanical properties compared to hardwood species monoculture plantations may reduce biodiversity potential for water depletion and soil degradation some species may require adhesive and pressing parameters adjustments for better bonding properties
Alternative non-plantation wood species	broad species diversity in both temperate and tropical zones potential of utilizing residues and pruning waste lower environmental impact than for plantation-based sourcing fast growth of some species some species (beech, birch) improve IB, surface soundness and stability fruit wood species meet or exceed standard requirements tropical species mixtures can enhance mechanical strength use of local species can support regional economies and reduce transport enables diversification of wood sources in the wood-based board industry	high variability in raw material quality non-uniform structure and particle behaviour in some cases, higher cutting resistance—increase tool wear seasonal or regionally limited availability limited large-scale industrial testing and adaptation some dense species show insufficient MOR and MOE requires sustainable forest management to prevent ecosystems degradation may require adjustments to resin systems and pressing parameters
Recycled wood	reduces demand for virgin timber supports circular economy and waste reduction diverts wood waste from landfills and incineration helps meet environmental regulation and green deal targets available and cost-effective feedstock enables partial substitution without quality loss encourages innovation in adhesives and grading systems	variable physical and chemical composition decreased mechanical properties at high substitution levels potential presence of contaminants (paints, adhesives, biocides) reduced bond strength challenges in processing and sorting mixed waste lower biological and fire resistance in some cases often limited to core layers or non-structural applications requires investment in pre-treatment and separation technologies

**Table 2:** Selected properties of PB and OSB boards made of various types of wood raw materials.

Kind of Raw Material	Type of Board	Kind of Adhesive	Density of Board	IB	MOR	MOE	WA	TS	Ref.
			kg/m^3^	MPa	MPa	MPa	%	%	
Juvenile wood									
Rubberwood clones RRIM 2002	PB	UF	700	1.40	22.5	2373	65.4	22.7	[52]
Pine	PB	UF	550	0.39	9.02	921	145.0 ^2h^	44.9 ^2h^	[54]
Silver birch				0.25	9.96	1466	113.3 ^2h^	40.5 ^2h^	
Radiata pine	OSB—100% JW OSB—70% JW	PF	650	0.46	34.4	3271	-	32.0	[56]
				0.65	38.7	4907	-	27.0	
Adler European larch poplar willow	OSB	pMDI	685	0.80	34.3	4472	21.4	48.2	[61]
			731	0.65	25.9	4410	12.0	32.9	
			680	0.65	25.2	3815	13.9	50.9	
			745	0.67	27.8	3939	10.5	43.0	
Wood from fast-growing plantation species									
Willow	PB	UF	680	1.44	17.8	2856	~70	~24.5	[73]
Poplar				2.02	21.5	~3000	~70	~24.5	
Willow	OSB	pMDI	610	1.33	36.6	4636	-	-	[84]
Poplar				1.28	43.3	3147	-	-	
Paulownia	PB	UF	500	~0.91	~9.0	~1450	~68	~13	[95]
	OSB	UF	700	~0.44	19.4	2350	70	40	[101]
Eucalyptus	PB	UF	867	0.63	12.4	1937	-	62	[111]
Native hardwood species									
Beech	PB—30% B	UF	645	0.38	8.6	2125	36.1	70.9	[133]
Apple	PB	UF	700	0.81	12.84	2432	19.6	-	[137]
Plum				0.77	14.38	2576	22.3		
Birch	OSB	pMDI	610	1.05	36.0	5665	-	-	[84]
Tropical fast-growing species									
Cambará	OSB	PU	650	0.66	30.20	5463	8.78	21.21	[139]
Paricá				0.54	52.90	6932	28.74	57.63	
Recycled wood									
Wood recycled from mixed demolition and construction (CDW)	PB—25% CDW	UF	750	0.83	5.67	1150	59.21	21.52	[148]
	PB—50% CDW			0.81	6.95	1206	72.51	24.64	
Wood recycled from window frames	PB—25% WF	UF	670	0.17	11.3	2357	89.5	45.0	[150]
	PB—50% WF			0.19	12.3	2511	87.1	39.3	
	PB—100% WF			0.47	14.5	3032	85.7	38.9	
Wood recycled from raw faulty PB	PB	UF	650	0.55	9.30	2194	33.46	9.72	[157]
Wood recycled from laminated PB	PB—20% RW	UF	700	0.34	13.4	-	-	18.8	[159]
	PB—60% RW			0.33	12.3			17.2	
Wood recycled from pallets	PB—20% RP	UF	650	0.70	12.1	2471	50.95	18.67	[162]
	PB—50% RP			0.68	12.4	2276	76.80	27.87	
	PB—100% RP			0.61	10.0	2012	56.77	23.67	

Note: JW—juvenile wood; B—beech; CDW—construction and demolition wood; WF—window frames; RW—recycled wood; RP—recycled pallets; IB—internal bond; MOR—bending strength; MOE—modulus of elasticity; WA—water absorption; TS—thickness swelling, ^2h^—sample soaking time (in hours); UF—urea–formaldehyde resin; PF—phenol–formaldehyde resin; pMDI—diphenylmethane diisocyanate; PU—polyurethane resin.

## Data Availability

Not applicable.
